# *N*-(Pyridin-2-ylmeth­yl)pyridin-2-amine

**DOI:** 10.1107/S1600536811016874

**Published:** 2011-05-07

**Authors:** Suk-Hee Moon, Tae Ho Kim, Ki-Min Park

**Affiliations:** a Department of Food & Nutrition, Kyungnam College of Information and Technology, Busan 616-701, Republic of Korea; b Department of Chemistry and Research Institute of Natural Sciences, Gyeongsang National University, Jinju 660-701, Republic of Korea

## Abstract

The title compound, C_11_H_11_N_3_, crystallizes with two mol­ecules (*A* and *B*) in the asymmetric unit. The geometries of both mol­ecules are very similar, with the exception of the torsion angles of the inter-ring chains; the values for C—N—C—C are 67.4 (5) and −69.3 (5)° for mol­ecules *A* and *B*, respectively. The dihedral angles between the pyridyl ring planes are 84.0 (2) and 83.2 (2)° for mol­ecules *A* and *B*, respectively. In the crystal, weak inter­molecular N—H⋯N hydrogen bonds and C—H⋯π inter­actions contribute to the stabilization of the packing.

## Related literature

For details of the synthesis, see: Foxon *et al.* (2002 ▶). For the crystal structures of Cu complexes of the title compound, see: Lee *et al.* (2008 ▶).
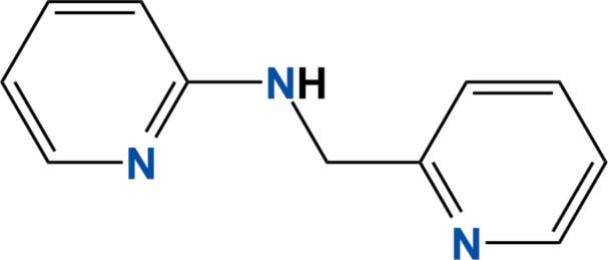


## Experimental

### 

#### Crystal data

C_11_H_11_N_3_
*M* *_r_* = 185.23
Orthorhombic, 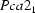

*a* = 14.5434 (14) Å
*b* = 5.8198 (6) Å
*c* = 23.045 (2) Å
*V* = 1950.5 (3) Å^3^
*Z* = 8
Mo *K*α radiation
μ = 0.08 mm^−1^
*T* = 173 K
0.45 × 0.30 × 0.30 mm

#### Data collection

Bruker APEXII CCD diffractometer
11034 measured reflections
2182 independent reflections
1814 reflections with *I* > 2σ(*I*)
*R*_int_ = 0.060

#### Refinement

*R*[*F* ^2^ > 2σ(*F* ^2^)] = 0.054
*wR*(*F* ^2^) = 0.124
*S* = 1.10
2182 reflections
253 parameters
1 restraint
H-atom parameters constrained
Δρ_max_ = 0.20 e Å^−3^
Δρ_min_ = −0.26 e Å^−3^

### 

Data collection: *APEX2* (Bruker, 2006 ▶); cell refinement: *SAINT* (Bruker, 2006 ▶); data reduction: *SAINT*; program(s) used to solve structure: *SHELXTL* (Sheldrick, 2008 ▶); program(s) used to refine structure: *SHELXTL*; molecular graphics: *SHELXTL* and *DIAMOND* (Brandenburg, 1998 ▶); software used to prepare material for publication: *SHELXTL*.

## Supplementary Material

Crystal structure: contains datablocks global, I. DOI: 10.1107/S1600536811016874/wn2432sup1.cif

Structure factors: contains datablocks I. DOI: 10.1107/S1600536811016874/wn2432Isup2.hkl

Supplementary material file. DOI: 10.1107/S1600536811016874/wn2432Isup3.cml

Additional supplementary materials:  crystallographic information; 3D view; checkCIF report

## Figures and Tables

**Table 1:** Hydrogen-bond geometry (Å, °) *Cg*1, *Cg*2 and *Cg*3 are the centroids of the N4/C12–C16, N2/C7–C11 and N5/C18–C22 rings, respectively.

*D*—H⋯*A*	*D*—H	H⋯*A*	*D*⋯*A*	*D*—H⋯*A*
N3—H3*N*⋯N4	0.89	2.14	3.019 (5)	172
N6—H6*N*⋯N1	0.93	2.10	3.012 (5)	168
C1—H1⋯*Cg*1^i^	0.95	2.77	3.53	137
C3—H3⋯*Cg*2^ii^	0.95	2.85	3.69	147
C14—H14⋯*Cg*3^iii^	0.95	2.65	3.51	149

Symmetry codes: (i) 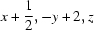; (ii) 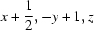; (iii) 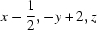.

**Fig. 1.**
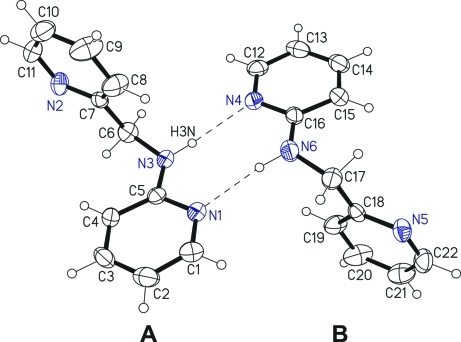
The structure of the asymmetric unit of the title compound, showing displacement ellipsoids drawn at the 50% probability level. Dashed lines indicate hydrogen bonds

**Fig. 2.**
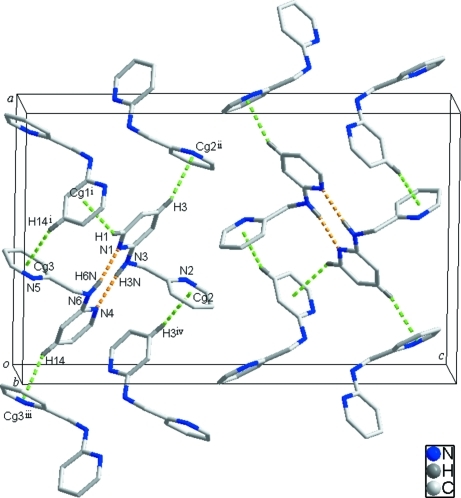
Crystal packing of the title compound with intermolecular N—H···N hydrogen bonds and C—H···π interactions shown as dashed lines. H atoms not involved in intermolecular interactions have been omitted for clarity. Cg1 Cg2 and Cg3 are the centroids of the N4/C12–C16, N2/C7–C11 and N5/C18–C22 rings, respectively. (Symmetry codes: i) x + 1/2, -y + 2, z; ii) x + 1/2, -y + 1, z; iii) x - 1/2, -y + 2, z; iv) x - 1/2, -y + 1, z)

## supplementary crystallographic information

### Comment

The title compound was prepared for use as a multidentate ligand in the formation of metallosupramolecules according to a published literature procedure (Foxon *et al.*, 2002). The crystal structures of Cu complexes of the title compound have already been reported (Lee *et al.*, 2008). However the crystal structure of the free form has not yet been reported.

The title compound (Scheme, Fig. 1) crystallized in the non-centrosymmetric space group *Pca*2_1_. The asymmetric unit contains two crystallographically independent molecules (A and B). The geometries of both molecules are very similar, with the exception of the torsion angles of the inter-ring chains; the value for C5—N3—C6—C7 is 67.4 (5) ° and for C16—N6—C17—C18 is -69.3 (5) °. The dihedral angles between the pyridyl ring planes are 84.0 (2)° and 83.2 (2) ° for molecules A and B, respectively.

In the crystal structure, the amine groups of both molecules are involved in pair-wise intermolecular N—H···N interactions, leading to the formation of dimers (Table 1, Fig. 1, Fig. 2). Weak intermolecular C—H···π interactions are also present (Fig. 2). These intermolecular interactions contribute to the stabilization of the packing.

### Experimental

The title compound was synthesized according to a literature procedure (Foxon *et al.*, 2002). Slow evaporation of a solution in CH_3_OH gave single crystals suitable for X-ray analysis.

### Refinement

All H atoms except those of the amine groups were positioned geometrically and refined using a riding model, with d(C—H) = 0.95 Å for Csp^2^—H and 0.99 Å for methylene C—H. H atoms of the amine groups were located in difference electron density maps and then refined using a riding model with N—H = 0.89 Å and 0.93 Å. For all H atoms *U*_iso_(H) = 1.2*U*_eq_(C,N). In the absence of significant anomalous scattering effects, Friedel pairs were merged.

### Crystal data

**Table:**

C_11_H_11_N_3_	*F*(000) = 784
*M**_r_* = 185.23	*D*_x_ = 1.262 Mg m^−^^3^
Orthorhombic, *P**c**a*2_1_	Mo *K*α radiation, λ = 0.71073 Å
Hall symbol: P 2c -2ac	Cell parameters from 4097 reflections
*a* = 14.5434 (14) Å	θ = 2.3–27.2°
*b* = 5.8198 (6) Å	µ = 0.08 mm^−^^1^
*c* = 23.045 (2) Å	*T* = 173 K
*V* = 1950.5 (3) Å^3^	Block, colorless
*Z* = 8	0.45 × 0.30 × 0.30 mm

### Data collection

**Table:**

Bruker APEXII CCD diffractometer	1814 reflections with *I* > 2σ(*I*)
Radiation source: fine-focus sealed tube	*R*_int_ = 0.060
graphite	θ_max_ = 27.0°, θ_min_ = 2.8°
φ and ω scans	*h* = −18→18
11034 measured reflections	*k* = −7→7
2182 independent reflections	*l* = −21→29

### Refinement

**Table:**

Refinement on *F*^2^	Primary atom site location: structure-invariant direct methods
Least-squares matrix: full	Secondary atom site location: difference Fourier map
*R*[*F*^2^ > 2σ(*F*^2^)] = 0.054	Hydrogen site location: inferred from neighbouring sites
*wR*(*F*^2^) = 0.124	H-atom parameters constrained
*S* = 1.10	*w* = 1/[σ^2^(*F*_o_^2^) + (0.0449*P*)^2^ + 1.1368*P*] where *P* = (*F*_o_^2^ + 2*F*_c_^2^)/3
2182 reflections	(Δ/σ)_max_ < 0.001
253 parameters	Δρ_max_ = 0.20 e Å^−^^3^
1 restraint	Δρ_min_ = −0.26 e Å^−^^3^

### Special details

**Table:**

Geometry. All e.s.d.'s (except the e.s.d. in the dihedral angle between two l.s. planes) are estimated using the full covariance matrix. The cell e.s.d.'s are taken into account individually in the estimation of e.s.d.'s in distances, angles and torsion angles; correlations between e.s.d.'s in cell parameters are only used when they are defined by crystal symmetry. An approximate (isotropic) treatment of cell e.s.d.'s is used for estimating e.s.d.'s involving l.s. planes.
Refinement. Refinement of *F*^2^ against ALL reflections. The weighted *R*-factor *wR* and goodness of fit *S* are based on *F*^2^, conventional *R*-factors *R* are based on *F*, with *F* set to zero for negative *F*^2^. The threshold expression of *F*^2^ > σ(*F*^2^) is used only for calculating *R*-factors(gt) *etc*. and is not relevant to the choice of reflections for refinement. *R*-factors based on *F*^2^ are statistically about twice as large as those based on *F*, and *R*- factors based on ALL data will be even larger.

### Fractional atomic coordinates and isotropic or equivalent isotropic displacement parameters (Å^2^)

**Table:**

	*x*	*y*	*z*	*U*_iso_*/*U*_eq_	
N1	0.4906 (2)	0.8350 (5)	0.22165 (14)	0.0310 (7)	
N2	0.3345 (2)	0.2870 (6)	0.38908 (15)	0.0372 (8)	
N3	0.4145 (3)	0.5075 (5)	0.24813 (15)	0.0324 (8)	
H3N	0.3691	0.5445	0.2242	0.039*	
C1	0.5596 (3)	0.9839 (7)	0.2290 (2)	0.0361 (10)	
H1	0.5633	1.1100	0.2029	0.043*	
C2	0.6248 (3)	0.9682 (8)	0.2711 (2)	0.0416 (12)	
H2	0.6728	1.0781	0.2741	0.050*	
C3	0.6183 (3)	0.7845 (8)	0.30973 (18)	0.0398 (10)	
H3	0.6617	0.7693	0.3403	0.048*	
C4	0.5493 (3)	0.6254 (7)	0.30356 (17)	0.0346 (9)	
H4	0.5449	0.4978	0.3291	0.041*	
C5	0.4854 (2)	0.6557 (6)	0.25870 (16)	0.0273 (8)	
C6	0.3857 (3)	0.3394 (6)	0.29081 (18)	0.0332 (9)	
H6A	0.4400	0.2470	0.3021	0.040*	
H6B	0.3411	0.2340	0.2723	0.040*	
C7	0.3424 (2)	0.4366 (6)	0.34538 (18)	0.0256 (8)	
C8	0.3101 (3)	0.6580 (6)	0.3489 (2)	0.0435 (11)	
H8	0.3184	0.7623	0.3176	0.052*	
C9	0.2650 (4)	0.7265 (7)	0.3993 (2)	0.0528 (13)	
H9	0.2412	0.8779	0.4026	0.063*	
C10	0.2553 (3)	0.5742 (7)	0.4439 (2)	0.0421 (11)	
H10	0.2245	0.6160	0.4787	0.050*	
C11	0.2916 (3)	0.3591 (8)	0.43671 (19)	0.0424 (10)	
H11	0.2857	0.2537	0.4680	0.051*	
N4	0.2551 (2)	0.6738 (5)	0.17549 (13)	0.0287 (7)	
N5	0.4108 (2)	1.2720 (5)	0.01448 (14)	0.0331 (7)	
N6	0.3312 (3)	1.0014 (5)	0.14957 (16)	0.0347 (9)	
H6N	0.3770	0.9641	0.1761	0.042*	
C12	0.1866 (3)	0.5246 (7)	0.1674 (2)	0.0336 (10)	
H12	0.1807	0.4016	0.1943	0.040*	
C13	0.1236 (3)	0.5373 (7)	0.1227 (2)	0.0396 (11)	
H13	0.0763	0.4260	0.1184	0.048*	
C14	0.1323 (3)	0.7189 (8)	0.08444 (18)	0.0417 (10)	
H14	0.0906	0.7338	0.0529	0.050*	
C15	0.2011 (3)	0.8777 (7)	0.09179 (17)	0.0359 (9)	
H15	0.2072	1.0033	0.0657	0.043*	
C16	0.2625 (2)	0.8514 (6)	0.13870 (17)	0.0282 (8)	
C17	0.3582 (3)	1.1827 (6)	0.11029 (17)	0.0323 (8)	
H17A	0.4007	1.2869	0.1311	0.039*	
H17B	0.3027	1.2728	0.1001	0.039*	
C18	0.4040 (2)	1.1067 (6)	0.05466 (17)	0.0255 (8)	
C19	0.4391 (3)	0.8888 (6)	0.0460 (2)	0.0381 (10)	
H19	0.4316	0.7733	0.0747	0.046*	
C20	0.4848 (3)	0.8417 (7)	−0.0045 (2)	0.0487 (12)	
H20	0.5108	0.6939	−0.0106	0.058*	
C21	0.4930 (4)	1.0085 (8)	−0.0462 (2)	0.0458 (12)	
H21	0.5244	0.9797	−0.0816	0.055*	
C22	0.4541 (3)	1.2192 (7)	−0.03478 (18)	0.0387 (9)	
H22	0.4583	1.3345	−0.0639	0.046*	

### Atomic displacement parameters (Å^2^)

**Table:**

	*U*^11^	*U*^22^	*U*^33^	*U*^12^	*U*^13^	*U*^23^
N1	0.0295 (17)	0.0376 (17)	0.0260 (17)	−0.0019 (14)	0.0012 (13)	0.0025 (14)
N2	0.043 (2)	0.0342 (17)	0.0342 (18)	0.0037 (15)	−0.0029 (16)	0.0128 (15)
N3	0.030 (2)	0.0433 (18)	0.024 (2)	−0.0028 (14)	0.0009 (17)	0.0062 (14)
C1	0.032 (2)	0.037 (2)	0.040 (3)	−0.0005 (16)	0.005 (2)	−0.0010 (18)
C2	0.028 (2)	0.053 (3)	0.044 (3)	−0.0048 (19)	0.002 (2)	−0.008 (2)
C3	0.024 (2)	0.061 (3)	0.034 (2)	0.0052 (18)	−0.0063 (17)	−0.005 (2)
C4	0.0283 (19)	0.050 (2)	0.025 (2)	0.0041 (18)	0.0005 (16)	0.0060 (18)
C5	0.0246 (18)	0.0343 (18)	0.0230 (18)	0.0020 (15)	0.0050 (15)	−0.0028 (16)
C6	0.035 (2)	0.0266 (17)	0.038 (2)	−0.0008 (16)	−0.0008 (18)	0.0007 (18)
C7	0.0192 (17)	0.0283 (17)	0.0292 (19)	−0.0044 (14)	−0.0030 (16)	0.0050 (15)
C8	0.047 (3)	0.0277 (19)	0.056 (3)	0.0024 (18)	0.016 (2)	0.014 (2)
C9	0.056 (3)	0.033 (2)	0.069 (3)	0.000 (2)	0.026 (3)	−0.003 (2)
C10	0.039 (2)	0.052 (2)	0.036 (3)	−0.008 (2)	0.009 (2)	−0.008 (2)
C11	0.043 (2)	0.055 (2)	0.029 (2)	0.001 (2)	0.0033 (19)	0.011 (2)
N4	0.0276 (17)	0.0320 (15)	0.0265 (17)	0.0025 (13)	0.0021 (13)	0.0006 (13)
N5	0.0383 (18)	0.0299 (16)	0.0310 (17)	0.0081 (14)	−0.0028 (15)	0.0027 (15)
N6	0.032 (2)	0.0382 (18)	0.034 (2)	−0.0037 (14)	−0.0074 (19)	0.0087 (15)
C12	0.028 (2)	0.0363 (19)	0.037 (2)	0.0048 (16)	0.009 (2)	−0.0018 (17)
C13	0.025 (2)	0.051 (2)	0.044 (3)	0.0010 (18)	0.001 (2)	−0.012 (2)
C14	0.027 (2)	0.067 (3)	0.031 (2)	0.0088 (19)	−0.0016 (17)	−0.004 (2)
C15	0.0261 (19)	0.048 (2)	0.033 (2)	0.0044 (17)	−0.0009 (17)	0.0066 (19)
C16	0.0249 (18)	0.0361 (18)	0.0237 (18)	0.0085 (15)	0.0019 (15)	0.0006 (16)
C17	0.035 (2)	0.0317 (19)	0.0303 (19)	0.0053 (16)	0.0031 (17)	0.0011 (17)
C18	0.0223 (16)	0.0235 (16)	0.0306 (19)	−0.0014 (15)	−0.0085 (16)	0.0005 (16)
C19	0.034 (2)	0.0250 (18)	0.055 (3)	0.0012 (16)	0.011 (2)	0.0082 (19)
C20	0.047 (3)	0.0262 (19)	0.073 (3)	0.0009 (18)	0.022 (2)	−0.008 (2)
C21	0.042 (3)	0.052 (2)	0.043 (3)	−0.0035 (19)	0.012 (2)	−0.014 (2)
C22	0.045 (2)	0.046 (2)	0.0252 (19)	−0.0010 (19)	−0.0057 (19)	0.0028 (18)

### Geometric parameters (Å, °)

**Table:**

N1—C1	1.336 (5)	N4—C12	1.335 (5)
N1—C5	1.350 (5)	N4—C16	1.341 (5)
N2—C11	1.331 (6)	N5—C22	1.334 (5)
N2—C7	1.337 (5)	N5—C18	1.339 (5)
N3—C5	1.365 (5)	N6—C16	1.351 (5)
N3—C6	1.449 (5)	N6—C17	1.445 (5)
N3—H3N	0.8867	N6—H6N	0.9301
C1—C2	1.360 (7)	C12—C13	1.382 (6)
C1—H1	0.9500	C12—H12	0.9500
C2—C3	1.394 (7)	C13—C14	1.382 (6)
C2—H2	0.9500	C13—H13	0.9500
C3—C4	1.373 (6)	C14—C15	1.373 (6)
C3—H3	0.9500	C14—H14	0.9500
C4—C5	1.401 (5)	C15—C16	1.410 (5)
C4—H4	0.9500	C15—H15	0.9500
C6—C7	1.516 (6)	C17—C18	1.511 (6)
C6—H6A	0.9900	C17—H17A	0.9900
C6—H6B	0.9900	C17—H17B	0.9900
C7—C8	1.374 (5)	C18—C19	1.381 (5)
C8—C9	1.391 (7)	C19—C20	1.367 (6)
C8—H8	0.9500	C19—H19	0.9500
C9—C10	1.365 (7)	C20—C21	1.372 (7)
C9—H9	0.9500	C20—H20	0.9500
C10—C11	1.368 (6)	C21—C22	1.376 (6)
C10—H10	0.9500	C21—H21	0.9500
C11—H11	0.9500	C22—H22	0.9500
			
C1—N1—C5	117.6 (3)	C12—N4—C16	118.2 (3)
C11—N2—C7	117.2 (3)	C22—N5—C18	117.2 (3)
C5—N3—C6	121.6 (3)	C16—N6—C17	123.8 (4)
C5—N3—H3N	121.3	C16—N6—H6N	120.1
C6—N3—H3N	111.7	C17—N6—H6N	112.9
N1—C1—C2	124.8 (4)	N4—C12—C13	124.4 (4)
N1—C1—H1	117.6	N4—C12—H12	117.8
C2—C1—H1	117.6	C13—C12—H12	117.8
C1—C2—C3	117.4 (4)	C12—C13—C14	117.1 (4)
C1—C2—H2	121.3	C12—C13—H13	121.4
C3—C2—H2	121.3	C14—C13—H13	121.4
C4—C3—C2	120.0 (4)	C15—C14—C13	120.2 (4)
C4—C3—H3	120.0	C15—C14—H14	119.9
C2—C3—H3	120.0	C13—C14—H14	119.9
C3—C4—C5	118.5 (4)	C14—C15—C16	118.9 (4)
C3—C4—H4	120.8	C14—C15—H15	120.6
C5—C4—H4	120.8	C16—C15—H15	120.6
N1—C5—N3	114.7 (3)	N4—C16—N6	116.1 (3)
N1—C5—C4	121.7 (3)	N4—C16—C15	121.2 (3)
N3—C5—C4	123.5 (3)	N6—C16—C15	122.7 (4)
N3—C6—C7	115.5 (3)	N6—C17—C18	115.9 (3)
N3—C6—H6A	108.4	N6—C17—H17A	108.3
C7—C6—H6A	108.4	C18—C17—H17A	108.3
N3—C6—H6B	108.4	N6—C17—H17B	108.3
C7—C6—H6B	108.4	C18—C17—H17B	108.3
H6A—C6—H6B	107.5	H17A—C17—H17B	107.4
N2—C7—C8	122.5 (4)	N5—C18—C19	122.2 (4)
N2—C7—C6	114.7 (3)	N5—C18—C17	114.1 (3)
C8—C7—C6	122.8 (4)	C19—C18—C17	123.7 (3)
C7—C8—C9	118.6 (4)	C20—C19—C18	119.1 (4)
C7—C8—H8	120.7	C20—C19—H19	120.5
C9—C8—H8	120.7	C18—C19—H19	120.5
C10—C9—C8	119.4 (4)	C19—C20—C21	119.8 (4)
C10—C9—H9	120.3	C19—C20—H20	120.1
C8—C9—H9	120.3	C21—C20—H20	120.1
C9—C10—C11	117.6 (4)	C20—C21—C22	117.4 (4)
C9—C10—H10	121.2	C20—C21—H21	121.3
C11—C10—H10	121.2	C22—C21—H21	121.3
N2—C11—C10	124.7 (4)	N5—C22—C21	124.2 (4)
N2—C11—H11	117.7	N5—C22—H22	117.9
C10—C11—H11	117.7	C21—C22—H22	117.9
			
C5—N1—C1—C2	0.0 (6)	N4—C12—C13—C14	−0.8 (6)
N1—C1—C2—C3	−0.8 (7)	C12—C13—C14—C15	−0.2 (6)
C1—C2—C3—C4	1.5 (6)	C13—C14—C15—C16	0.3 (6)
C2—C3—C4—C5	−1.3 (6)	C12—N4—C16—N6	178.1 (4)
C1—N1—C5—N3	−178.8 (4)	C12—N4—C16—C15	−1.6 (5)
C1—N1—C5—C4	0.2 (5)	C17—N6—C16—N4	170.6 (3)
C6—N3—C5—N1	−165.2 (3)	C17—N6—C16—C15	−9.7 (6)
C6—N3—C5—C4	15.8 (6)	C14—C15—C16—N4	0.6 (6)
C3—C4—C5—N1	0.5 (6)	C14—C15—C16—N6	−179.0 (4)
C3—C4—C5—N3	179.3 (4)	C16—N6—C17—C18	−69.3 (5)
C5—N3—C6—C7	67.4 (5)	C22—N5—C18—C19	−0.7 (6)
C11—N2—C7—C8	1.5 (6)	C22—N5—C18—C17	177.2 (3)
C11—N2—C7—C6	−175.8 (3)	N6—C17—C18—N5	166.9 (3)
N3—C6—C7—N2	−166.2 (3)	N6—C17—C18—C19	−15.2 (5)
N3—C6—C7—C8	16.5 (5)	N5—C18—C19—C20	2.1 (6)
N2—C7—C8—C9	−2.0 (7)	C17—C18—C19—C20	−175.6 (4)
C6—C7—C8—C9	175.1 (4)	C18—C19—C20—C21	−1.7 (7)
C7—C8—C9—C10	1.0 (8)	C19—C20—C21—C22	0.1 (7)
C8—C9—C10—C11	0.4 (7)	C18—N5—C22—C21	−1.1 (6)
C7—N2—C11—C10	0.0 (7)	C20—C21—C22—N5	1.4 (7)
C9—C10—C11—N2	−1.0 (7)	C5—N3—C6—C7	67.4 (5)
C16—N4—C12—C13	1.7 (6)	C16—N6—C17—C18	−69.3 (5)

### Hydrogen-bond geometry (Å, °)

**Table:**

Cg1, Cg2 and Cg3 are the centroids of the N4/C12–C16, N2/C7–C11 and N5/C18–C22 rings, respectively.

**Table:**

*D*—H···*A*	*D*—H	H···*A*	*D*···*A*	*D*—H···*A*
N3—H3N···N4	0.89	2.14	3.019 (5)	172
N6—H6N···N1	0.93	2.10	3.012 (5)	168
C1—H1···Cg1^i^	0.95	2.77	3.53	137
C3—H3···Cg2^ii^	0.95	2.85	3.69	147
C14—H14···Cg3^iii^	0.95	2.65	3.51	149

Symmetry codes: (i) *x*+1/2, −*y*+2, *z*; (ii) *x*+1/2, −*y*+1, *z*; (iii) *x*−1/2, −*y*+2, *z*.
